# Detection of Genomic Copy Number Variations in Ovarian Cancer in the Peripheral Blood System

**DOI:** 10.3390/cancers17050780

**Published:** 2025-02-25

**Authors:** Lena Wahl, Ulyana Hliabtsova, Xueqian Qian, Anastasia Klopf, Nina Hedemann, Inken Flörkemeier, Christoph Rogmans, Michael Kalab, Astrid Dempfle, Nicolai Maass, Dirk Olaf Bauerschlag, Marion Tina van Mackelenbergh, Jörg Paul Weimer, Huy Duc Le

**Affiliations:** 1 Department of Gynaecology and Obstetrics, Christian-Albrechts-University and University Medical Center Schleswig-Holstein, 24105 Kiel, Germany; 2 Institute of Medical Informatics and Statistics, Kiel University and University Medical Center Schleswig-Holstein, Campus Kiel, 24105 Kiel, Germany; 3 Clinic and Polyclinic for Gynecology and Reproductive Medicine, University Hospital Jena, 07747 Jena, Germany

**Keywords:** ddPCR, ovarian cancer, aCGH, CNV

## Abstract

Background/Objectives: The high mortality rate of ovarian cancer is due to its relative lack of symptoms. The earlier OC is detected, the more successful the treatment is. Here, we investigate whether cell-free tumor DNA (cftDNA) can be detected in peripheral blood by typical genome imbalances using digital droplet PCR (ddPCR). Methods: A dilution of DNA with known CNVs in an unremarkable diploid DNA simulates the detection of CNVs in cell free DNA (cfDNA) in blood plasma. Using aCGH, we detect typical genomic imbalances and regions with only less aberrations in 30 OC. This information is used to produce ddPCR tests that compare regions with high levels of imbalance to regions with low levels of imbalance. Further, 18 ddPCR tests were performed on plasma samples from 55 probands without tumor background and 15 patients with ovarian cancer before surgery. Results: Six ddPCR tests can significantly differentiate between subjects without tumor background and patients with OC. OC can be assumed with at least four abnormal ddPCR test results of these six significant tests. This evaluation detects OC with a specificity of 0.8 and a sensitivity of 0.8. Conclusions: Immediately before surgery, OC can be detected quite well by ddPCR in patients with a relatively high tumor burden. Whether this is also possible when the tumor burden is lower is currently being determined in another study of patients undergoing therapy. If this is confirmed, ddPCR offers a possibility for early detection.

## Simple Summary

Our research aims to test the suitability of ddPCR for the detection of tumor-related genomic imbalances (CNVs) in ovarian cancer (OC) in the peripheral blood system. In principle, in an artificial CNV simulation, we can show that CNVs can be detected even in larger dilutions in diploid DNA using ddPCR. Furthermore, we identified ovarian cancer CNVs by using aCGH and used this information to produce suitable ddPCR tests. These ddPCR tests are based on the principle of two differently colored comparative DNA loci that mark both very common genome imbalances in OC and regions that hardly indicate genome imbalances in OC. We were able to generate six ddPCR tests that indicate OC by identifying cell-free tumor DNA with a sensitivity of 0.8 and a specificity of 0.8.

## 1. Introduction

Ovarian cancer has an incidence of 11.8 per 100,000 women. It is responsible for about 5% of cancer-related deaths in women [1]. The early detection of ovarian cancer is associated with a better prognosis for the patient [2]. For decades, we have looked for typical markers that allow us to detect ovarian cancer (OC) [3,4,5,6]. In our understanding of biological processes, we naturally look for a comprehensible, rational process. Oncogenes, driver mutations, and defective tumor suppressors fit well into our understanding. The development of novel new-generation sequencing (NGS) panels, which are just beginning, is increasing the expectation for truly promising indicators [7]. Although an effective biomarker for OC is still a long way off, predictive biomarkers for OC exist: the detection of *BRCA* mutations is beneficial for patients with OC if they can then be treated with a PARP inhibitor [8]. Our efforts are accompanied by ever better, more sophisticated, and more expensive technology. We now sequence entire tumor genomes in search of relevant mutations that allow us to offer patients individualized therapy. These efforts are certainly worthwhile and justified, especially if they ultimately help the patient. However, these markers usually only take effect when a diagnosis of OC is already available. However, a marker is needed to indicate OC before a patient presents to a doctor with symptoms and is taken for surgery. Let us examine whether the search for early detection markers must necessarily go hand in hand with our understanding of the ratio of oncogenesis. We have known for a quarter of a century that the genomes of solid tumors more or less exhibit pronounced genomic chaos [9]. So far, we have searched for genes relevant to oncogenesis in this chaos in order to be able to derive therapeutic approaches. In this study, we tried to use the detection of genetic chaos itself as an independent marker. Modern techniques such as digital PCR allow for comparative digital droplet PCR (ddPCR) to detect copy number variations (CNVs), for example in solid tumors in the peripheral blood. The advantage of this technique is the detectability of even the smallest amounts of DNA, down to single molecules. As Heng et al. (2021) have already shown, the events and genetic changes that occur in the cells of solid tumors during chromoangiogenesis are a very concentrated process that occurs within a few cell cycles [10,11]. Chromothripsis leads to a sudden separation of the genome in the chromatids during anaphase [12,13,14,15]. Chromoanasynthesis results from errors in chromosome replication, leading to an accumulation of restructuring and rearrangements within a chromosome [16,17]. And thirdly, frequent chromosome rearrangements of the chromoplexy in the G1 and G2 phases lead to complex chromosome rearrangements. All of these changes occur within a few events and are typical for most solid tumors. The detectable complex genome restructuring in solid tumors is a consequence of these processes. As a result, such solid tumor cells exhibit genomic imbalances that deviate significantly from a diploid state. Technical developments, particularly in the detection of very few residual traces of these genomic imbalances, now allow us to detect these tumor-typical traces as cell free DNA (cfDNA) in the peripheral blood [18,19]. Digital droplet PCR allows for the detection of CNVs even with only a few single molecules in the plasma or serum [20], compared with genomic loci that rarely indicate genomic alterations as a reference.

**Figure 1:**
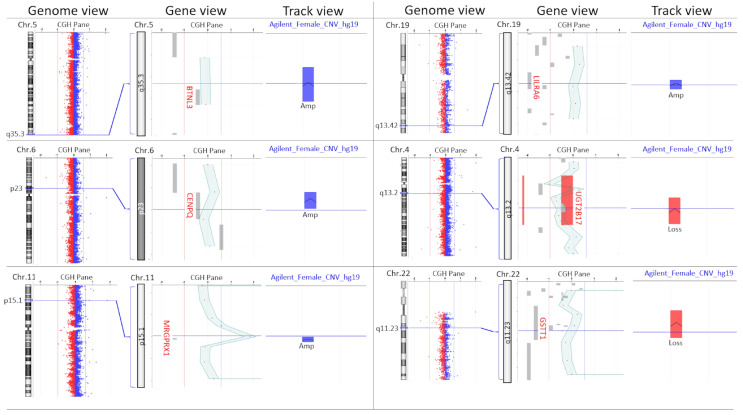
Microarray-based Comparative Genomic Hybridization (aCGH) profile of Agilent human reference female DNA compared to inconspicuous pool Kreatech reference DNA on a 4x180k SurePrint G3 Cancer CGH+SNP array (design: 030587) from Agilent. Six known loci are shown to exhibit imbalances in the Agilent human reference female DNA. On the left, the genome view is visible next to the corresponding chromosome ideogram. In the middle, the aCGH profile is visible in the gene view with corresponding genes. The red vertical line marks the limit of a significant loss and the light blue line marks the limit of a significant gain. On the right side, the track view shows the known gains (blue) and the known losses (red) in the Agilent human reference female DNA.

The aim of this study is to initially test whether a comparative ddPCR is suitable for detecting CNVs, even in greater dilution. Furthermore, we want to use OC-specific ddPCR tests to test whether CNVs in cfDNA can be detected using this method. In this study, we checked whether comparative ddPCR is suitable for detecting tumor genomic imbalances, which should be present as a trace of cell-free DNA in the blood. ddPCR has the advantage of dividing the reaction volume into 18–25 thousand reaction volumes. This means that individual molecules already present a detectable product within a reduced reaction volume, such as a droplet. This means that CNVs can even be detected at concentrations as low as those required for PCR reactions. First, we simulated artificial CNVs in different mixing ratios with unremarkable diploid DNA and found out whether these known CNVs could be detected at all using ddPCR against the background of diploid DNA from normal cells. Known amplifications and known deletions in the Agilent human reference female DNA (Figure 1) were diluted in a reference pool DNA mixture from 100 people (Kreatech; https://www.diagnostictechnology.com.au/products/megapool-reference-dna-female-100-individuals, accessed on 1 October 2022). Serial dilutions of CNV-bearing Agilent DNA in diploid Kreatech DNA were performed to demonstrate the limits of detection of amplifications and deletions. Loci with known CNVs were labeled with a Taq-Man test dye FAM. Inconspicuous locus were labeled with a HEX dye.

**Figure 2:**
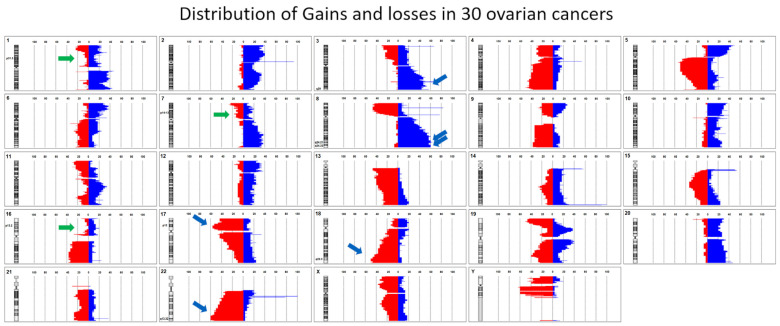
The percentage of gains and losses in 30 OCs analyzed by aCGH. The ideograms of the chromosomes are shown on the left. Red bars show losses and blue bars show gains. The green arrows show barely changed regions that show hardly any imbalances in OC. The blue arrows mark loci with the most gains and losses.

In order to be able to use this method to detect cell-free DNA from a tumor (cftDNA), such as that of OC, typical markers that can identify OC are first required. The identification of frequently recurring genome imbalances in OC was carried out by array-based comparative genome hybridization (aCGH) on 30 primary OC tumors. In a further step, we identified the loci that most frequently showed deviations from the diploid, balanced status in OC and the loci that showed the fewest deviations (Figure 2). We have already published these data in Limburg et al. 2024 [21]. The loci 18q22.3; 17p12, and 22q13.32 showed losses in the tumor genome in 50–60% of the OC primary tumors examined. In the loci 8q24.21, 8q24.22, and 3q29, genomic gains were detectable in 56–63% of the OC primary tumors examined. The regions 1p31.3, 7p14-13, and 16p13.2 were rather unremarkable in the OC tumors, appearing in only 15–25% of cases. The detection of conspicuous loci using ddPCR should differ quantitatively from that of inconspicuous loci in patients with tumor(s), whereas this should not be the case in healthy subjects. Each conspicuous locus was compared with an inconspicuous reference locus. In order to provide detection methods for the conspicuous loci for the ddPCR, we generated ddPCR tests for the conspicuous loci and marked them with the fluorophore FAM. For the more inconspicuous reference loci, we generated ddPCR tests that were marked with the fluorophore HEX.

For the purpose of this study, we applied the procedure in a blinded manner to 55 subjects without tumor background and 15 patients with OC before surgery. If the possibility of detecting genomic imbalances proves to be achievable both in the simulation and in the test cohort of patients with OC and normal subjects used here, then the applicability of this test as an early detection method must be assessed in a separate study.

## 2. Materials and Methods

### 2.1. DNA for Simulation Tests

The Agilent Human Reference female DNA (Agilent Part Number:5190-3797, Santa Clara, CA, USA) shows genomic gains in the regions 5q35.3 (*BTNL3*), 6p12.3 (*CENPQ*), 11p15.1 (*MRGPRX*), and 19q13.42 (*LILRA6*) (Figure 1). The Agilent Human Reference female DNA has genomic losses in the regions 4q13.2 (*UGT2B17*) and 22q11.23 (*GSTT1*). This Agilent Human Reference female DNA is mostly balanced in the regions 7p13-14 (*HECW1*), 1p31.3 (*JAK1*), and 16p13.2 (*USP7*). The Agilent Human Reference female DNA with the imbalances here described was used to investigate the performance of comparative ddPCR in dilutions with Kreatech Megapool Reference DNA (Kreatech, Amsterdam, The Netherlands). The Megapool reference DNA consisted of 100 genomes that, on average, had hardly any CNVs. The ddPCR CNV assays were provided in gene-specific form by BIORAD (Hercules, CA, USA) in such a way that CNVs of the Agilent human reference female DNA were marked with the fluorophore FAM and the inconspicuous, diploid areas were marked with the fluorophore HEX.

### 2.2. Array-Based Comparative Genomic Hybridization (aCGH)

We compared the Kreatech Megapool reference DNA with the Agilent human reference female DNA on a 4x180k SurePrint G3 Cancer CGH+SNP array (design: 030587) from Agilent. We followed the manufacturer’s instructions exactly. The array was read on a Dx microarray scanner G5761A from Agilent (Agilent, Santa Clara, CA, USA). The Agilent Feature Extraction Software (version 3.0.5.1, Agilent Technol. Inc., Santa Clara, CA, USA) and the Agilent Cytogenomics software (version 3.0.6.6, Agilent Technol. Inc., Santa Clara, CA, USA) were used for the evaluation.

### 2.3. Simulation of CNV Detection Using Dilutions of Agilent Human Reference Female DNA

First, the selected ddPCR CNV assays were compared in a dilution series from 1:2 to 1:128 with both the Agilent Human Reference female DNA and Kreatech Megapool Reference DNA. This was performed to select ddPCR CNV assays that can detect CNVs. The most suitable ddPCR tests were used with 1:2, 1:4, 1:8, 1:16, 1:32, 1:64, 1:128, and 1:25 dilutions of Agilent Human Reference female DNA in Kreatech Megapool Reference DNA. Each ddPCR-CNV assay (FAM) was performed simultaneously with an unremarkable reference ddPCR-CNV assay (HEX) in one run. The ddPCR-CNV assays were performed according to the manufacturer’s protocols.

### 2.4. ddPCR Tests

For the simulation, ddPCR CNV assays were provided by BioRad (Hercules, CA, USA). FAM-labeled ddPCR CNV assays for the genes *BTNL3* (assay ID: dHsaCNS655951788), *CENPQ* (assay ID: dHsaCNS868690000), *MRGPRX* (assay ID: dHsaCP1000474), and *LILRA6* (assay ID: dHsaCNS273681612) detected genome gains in the Agilent human reference female DNA. FAM-labeled ddPCR CNV assays of the genes *UGT2B17* (assay ID: dHsaCNS908621821) and *GSTT1* (assay ID: dHsaCP2500312) represented genome losses in the Agilent Human Reference female DNA. HEX-labeled ddPCR primers of the genes *HECW1* (assay ID: dHsaCNS351405409), *JAK1* (assay ID: dHsaCNS897218356), and *USP7* (assay ID: dHsaCNS806739955) amplified gene regions that are not subject to genomic changes in the Agilent Human Reference female DNA.

In order to be able to detect genomic imbalances in the cfDNA in OC after the simulation, ddPCR-CNV assays that correspond to the conspicuous regions typical of OC were ordered from BioRad. FAM-labeled ddPCR-CNV assays of the genes *ZFAT* in 8q24.22 (assay ID: dHsaCNS464948674), *PAK2* in 3q29 (assay ID: dHsaCNS290308003), and *PVT1* in 8q24.21 (assay ID: dHsaCNS911399324) correspond to the gains in OC. FAM-labeled ddPCR-CNV assays of the genes *Timm21* in 18q22.3 (assay ID: dHsaCNS263027692), *MYOCD* in 17p12 (assay ID: dHsaCNS286154155), and the region chr22q13.32 (assay ID: dHsaCNS366711081) correspond to the genomic losses in OC. HEX-labeled ddPCR-CNV assays of the genes *HECW1* in 7p14-13 (assay ID: dHsaCNS351405409), *JAK1* in1p31.3 (assay ID: dHsaCNS897218356), and *USP7* in 16p13.2 (assay ID: dHsaCNS806739955) show rare changes in OC. The combination of the FAM-labeled assays, which detect highly conspicuous OC loci, and the HEX-labeled assays, which identify less conspicuous loci, are intended to indicate the existence of cftDNA in OC when the number of detected FAM-positive droplets and HEX-positive droplets differ greatly.

### 2.5. Representation of cfDNA in Patients with OC and Subjects Without a Tumor Background

We took 8 mL of blood from 15 patients with OC before surgery and from 55 subjects without a tumor background using a Paxgene Blood ccfDNA Tube (PreAnalytiX, Hilden, Germany, Cat. No. 768115) and isolated the cell-free DNA using the QIAamp DSP Circulating NA Kit (Qiagene, Hilden, Germany) according to the manufacturer’s instructions. The blood sampling and its use for this research project was based on a vote by the Ethics Committee of the Medical Faculty of the Christian-Albrechts University of Kiel (D 420/23). The study participants were informed about the research project and agreed to it. The biosamples became the property of the BMB-GYN biosample bank, which is part of the p2n biobank network in Kiel. Before the ddPCR-CNV assays were carried out, the authors were blinded to the blood samples. Only after completion of the comparative ddPCR-CNV assays of all blood samples was it revealed whether a sample belonged to an OC patient or to a subject without tumor background.

### 2.6. Digital Droplet PCR (ddPCR)

The ddPCR was carried out in a volume of 22 µL with 1× ddPCR Supermix for Probes (No dUTP), 1× FAM ddPCR-CNV assay, 1× HEX ddPCR-CNV assay (BioRad, Hercules, CA, USA), 2 µL cfDNA, and 0.23 U/µL HAEIII restriction enzyme. Each comparative ddPCR test combination was carried out in technical triplets. The restriction digestion was incubated for 15 min at room temperature. In the QX200 Droplet Generator (BioRad, Hercules, CA, USA), the entire reaction volume was separated into small reaction drops according to the manufacturer’s protocol. After transferring the drops to a 96-well plate, the plate was sealed tightly with an adhesive-free film. This was followed by a PCR reaction in a C1000 Touch Thermal Cycler (BioRad, Hercules, CA, USA). The conditions were 10 min of enzyme activation at 95 °C, 30 s of DNA denaturation at 94 °C, and an increasing temperature ramp of 2 °C/second starting at 60 °C for 1 min. Then, this was repeated 49 times from the denaturation step. Finally, enzyme deactivation was carried out for 10 min at 98 °C. The product was finally cooled at 4 °C until evaluation. Droplets were read out in a QX200 Droplet Reader (BioRad, Hercules, CA, USA). The analysis software used was QuantaSoft Ver.1.2 (BioRad, Hercules, CA, USA).

### 2.7. Statistical Analysis

To evaluate the simulation, the absolute number of positive droplets was compared with the corresponding dilution of the template DNA and displayed as a graph.

To evaluate the blood samples, a quotient was formed from the number of drops with a positive FAM signal and the number of drops with a positive HEX signal. A mean value (Xi) was calculated from the technical triplets. From this averaged quotient, we subtracted the mean value (Y) of all quotients from all probands without a tumor background in the corresponding comparative ddPCR-CNV assay combination and divided it by the determined standard deviation (SD[Y]) from all probands without a tumor background in the corresponding comparative ddPCR-CNV assay combination. The thus normalized standard deviations (Xs) of the probands without a tumor background and Xs of the patients with OC were compared using a two-sample *t*-test (Origin, version 2021. OriginLab Corporation, Northampton, MA, USA). Only comparative ddPCR-CNV assay combinations whose value distribution differed significantly between subjects without tumor background and patients with OC with *t* < 0.01 were considered as suitable test combinations.

From the normalized standard deviation (Xs) of this suitable comparative ddPCR-CNV assay combination, the upper and lower 25% percentiles for each comparative ddPCR-CNV assay combination were determined from all probands without a tumor background (Origin, version 2021. OriginLab Corporation, Northampton, MA, USA). Values determined outside these percentile limits were considered abnormal. If the value determined from the blood of a test subject was outside the upper or lower 25% percentiles in at least four comparative ddPCR-CNV assay combinations, OC was assumed in the person examined. If there were fewer than four, this assumption was rejected. The selected ddPCR test combinations were subjected to a generalized cluster analysis on the 70 collective subjects. Statistica Version 14.0.1.25 (TIBCO Software Inc., Santa Clara, CA, USA) was used for this. Compared to tumor status, the six test combinations that showed the greatest significance in differentiation were identified and then confirmed using the best two-sample *t*-test.

## 3. Results

### 3.1. Simulation CNV Detection

**Figure 3:**
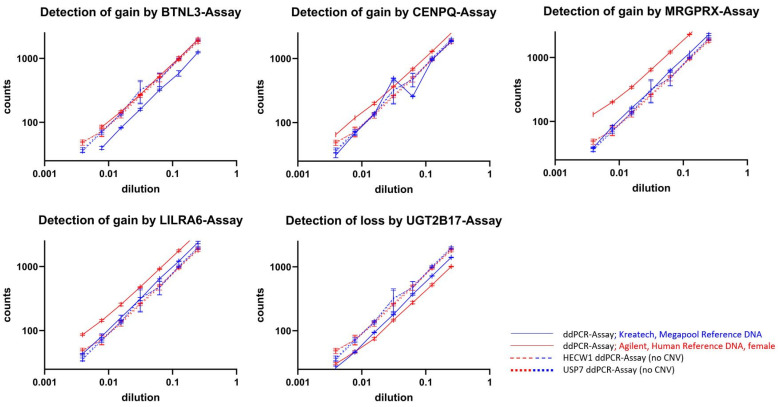
Here, the positively detected droplets are plotted against the degree of dilution. For the five functioning tests in the simulation, the positively detected droplets are plotted against the degree of dilution. The solid lines (blue = Kreatech Megapool; red = Agilent human reference female DNA) correspond to the respective tests that indicate imbalances in the Agilent human reference female DNA. The dotted lines show the tests that hardly indicate any imbalances in the Agilent human reference female DNA (blue = Kreatech Megapool; red = Agilent human reference DNA female).

**Figure 4:**
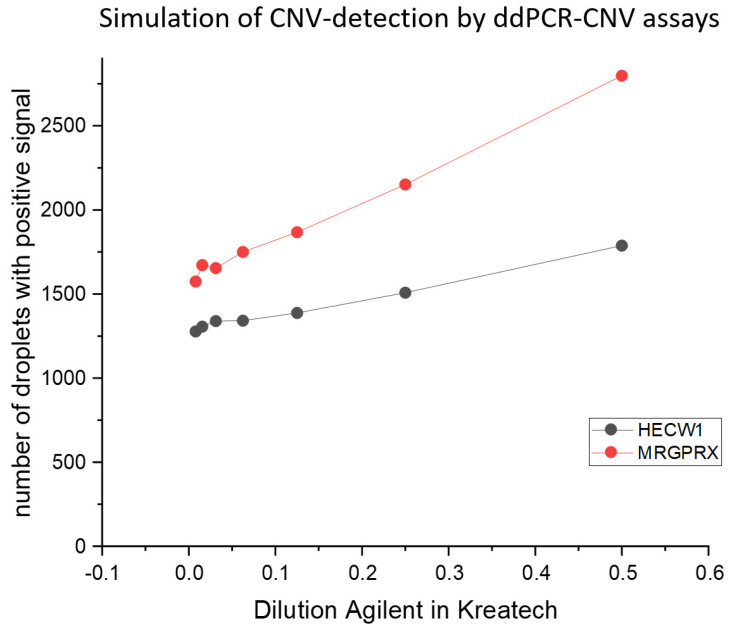
Here, the number of positive droplets is plotted against the dilution of the CNV-carrying Agilent human reference female DNA in diploid Kreatech pool DNA. The *MRGPRX* test always detects more positive droplets at all dilution levels than the *HECW1* test, in which a diploid state is present. Even at a 1:128 dilution (=0.0078125), the amplification in *MRGPRX* is still clearly detectable in this simulation.

Unfortunately, the experiments with the ddPCR-CNV assay for *GSTT1* did not produce any evaluable results. This assay probably has a fundamental malfunction. The ddPCR-CNV assay of the *BTNL3* region showed evaluable results in the dilution series of the Agilent Human Reference female DNA. However, the assay did not show an increased number of droplets with *BTNL3* signals compared to the inconspicuous regions of *HECW1* and *USP7* in the ddPCR-CNV assay as would be expected for a known overrepresentation. The frequency of these signals remained at the same level as that of *HECW1* and *USP7* in all dilution steps (Figure 3). Instead, the number of positive *BTNL3* droplets in all dilutions was lower than that of *HECW1* and *USP7* when Kreatech Megapool Reference DNA is used as the template. The ddPCR-CNV assay of the *CENPQ* region showed a higher number of positive droplets in most dilutions with the Agilent template. However, this was not the case in all. In all dilutions, the ddPCR-CNV assays of the *MRGPRX* and *LILRA6* regions showed more positive droplets than the comparisons with *HECW1* and *USP7* when Agilent Human Reference female DNA was used as the template. When the template was Kreatech Megapool Reference DNA, the number of droplets with positive signals remained at the same level as *HECW1* and *USP7*. Losses in the Agilent Human Reference female DNA were also detectable. *UGT2B17* was detectable up to a dilution of 1:32 when Agilent Human Reference female DNA was used as the template. With the Kreatech Megapool Reference DNA, the number of positive droplets remained the same. Since *MRGPRX* showed the clearest difference to the unremarkable *HECW1* and *USP7* ddPCR-CNV assays in these dilution tests, it is shown as an example in Figure 3; with this ddPCR-CNV assay, even a dilution of the Agilent DNA in Kreatech DNA of 1:128 still showed a clear difference to the unremarkable reference *HECW1*, although the unremarkable template was presented in excess (Figure 4).

### 3.2. Detection of Cell-Free Tumor DNA (cftDNA) in Peripheral Blood Using ddPCR-CNV Assays

**Figure 5:**
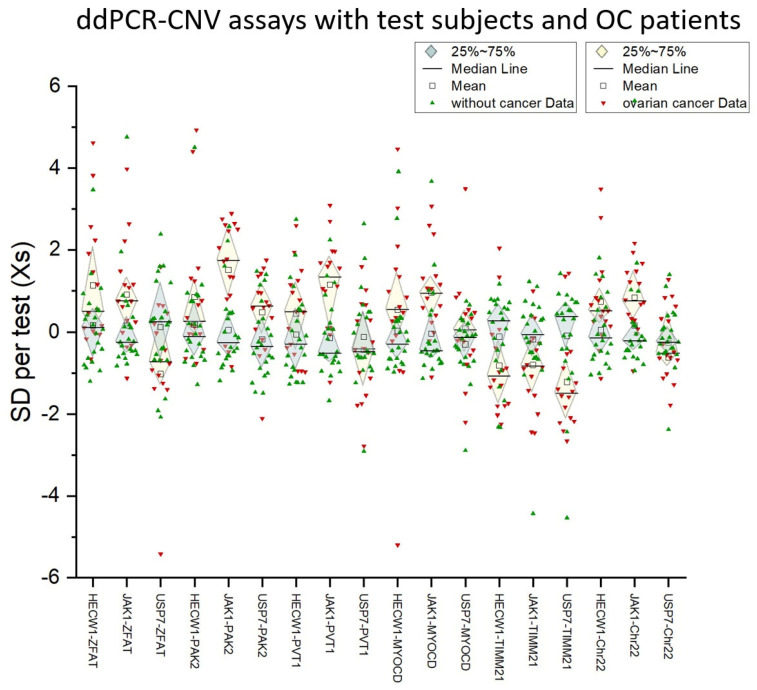
Here, the standard deviations (Xs) per ddPCR combination test of 55 probands without tumor background (green) and of 15 patients with OC (red) immediately before surgery are plotted.

cfDNA was successfully detected from 55 probands without a tumor background and from 15 patients with OC. The ddPCR-CNV assays were successfully performed on all cfDNA samples. Figure 5 shows the normalized standard deviations (Xs) of all subjects and patients examined in the ddPCR-CNV assay combinations. A *t*-test between the values of probands without a tumor background and patients with OC performed for each comparative ddPCR-CNV assay combination revealed the combinations that can identify genomic imbalances (Table 1). The selection of suitable ddPCR test combinations for the detection of cftDNA in the blood in OC was based solely on genomic loci with over- and underrepresentation, as well as inconspicuous loci as references in OC, which were determined by aCGH. The selected ddPCR test combinations were subjected to a cluster analysis using the Xs values of all patients (Supplementary Table S1). Of all 18 ddPCR test combinations, 14 test combinations showed significant discrimination in an ANOVA evaluation. Six of these discriminatory ddPCR test combinations confirmed this in a two-sample *t*-test with a *p*-value below 0.01 (Table 1). The results of the ANOVA (Supplementary Table S2) and a cluster analysis (Supplementary Table S3) are provided as additional material.

**Figure 6:**
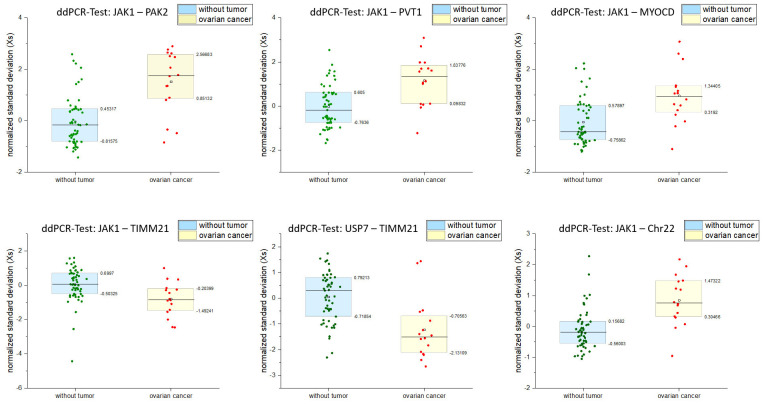
Here, the six ddPCR tests that clearly differentiate between subjects without tumor background and patients with OC are plotted.

Figure 6 compares the ddPCR-CNV assay combinations that significantly differentiated between probands without a tumor background and patients with OC. If the Xs values of a blood sample in at least four assays of these six significant ddPCR-CNV assay combinations (Xs) were outside the upper (75%) and lower (25%) percentiles determined from all probands without a tumor background (see Table 1), then it was assumed that cftDNA was detected as a typical genomic imbalance of OC.

In Table 2, we show the proportions of cases identified as true and false according to our test. The actual ovarian cancer cases are listed separately. Supplementary Table S1 shows the Xs values determined for the individual blood samples for each significant ddPCR-CNV assay combination. The Xs values that lie outside the permissible assay-specific percentiles are highlighted in yellow. In total, 44 of the 50 subjects without a tumor background were tested as truly normal. Of the 15 patients with OC, genomic imbalance in OC cells was detected as cftDNA in 12 blood samples. Three blood samples from patients with OC could not be identified as such. However, among all probands without a tumor background, 11 blood samples were falsely found to be abnormal. This results in a sensitivity of 0.8 and a specificity of 0.8 for this test. All the ddPCR assays whose values are outside the 25–75% percentiles of normal subjects without a tumor background are highlighted in yellow. If at least four Xs values are outside these 25–75% percentiles, the existence of OC is assumed.

## 4. Discussion

In the simulation, the direct comparison between Agilent and Kreatech templates regarding *BTNL3* signals initially agreed with expectations (Figure 3). In the Agilent template, more *BTNL3* calls were detected in all dilutions than in the Kreatech template. However, the supposedly increased *BTNL3* calls of the Agilent template were at the same level as the unremarkable *HECW1* and *USP7* reference tests in both templates, which corresponds to a diploid pattern. Consequently, *BTNL3* should be underrepresented in the Kreatech DNA pool of one hundred individuals. However, this is difficult to imagine in a pool of one hundred individuals. As Figure 1 shows, we were unable to confirm the amplification of the Agilent reference DNA female using aCGH as stated by Agilent (Figure 1). The neighboring signals of this supposed amplification look rather unremarkable in the aCGH. However, the resolution of the aCGH was rather low at this point. Whatever the cause of this surprising result, *BTNL3* should not be considered any further, just like the technical failure of *GSTT1*. The *CENPQ* assay also showed hardly any abnormalities in the aCGH (Figure 1), although here the resolution of this array was also rather low to be able to confirm the defined amplification in the Agilent reference DNA female. Nevertheless, the *CENPQ* ddPCR assay generally showed more ddPCR signals in the Agilent reference DNA female than in the unremarkable reference assays of *HECW1* and *USP7* (Figure 3). The outliers in the two dilutions of the Kreatech pool DNA may be related to pipetting errors. However, the cause is not relevant at this point. This is because the two assays for *LILRA6* and *MRGPRX* confirm the existence of defined amplification in the Agilent human reference female DNA across all the dilutions tested. Meanwhile, the number of positive calls in these *LILRA6* and *MRGPRX* assays in the Megapool Kreatech reference DNA is at the same level as in the unremarkable diploid *HECW1* and *USP7* assays (Figure 3). Although the resolution of the aCGH is barely sufficient to confirm the defined amplification of *LILRA6*, the defined amplification of *MRGPRX* in the Agilent reference DNA female template is also clearly visible in the aCGH (Figure 1). Even at the highest dilution, both assays clearly show amplification in Agilent reference DNA female. Since *GSTT1* failed technically, only the *UGT2B17* assay remains, with which we could test for evidence of genomic loss. We were also able to confirm the defined genomic loss in Agilent reference DNA female with the aCGH (Figure 1). This loss in *UGT2B17* is clearly detectable up to a dilution of 1:32. Only at even higher dilutions can the test no longer indicate the genomic loss (Figure 3). The performance limit of our best discriminating assay for amplifications was tested in the simulation by a dilution series of the Agilent reference DNA female in the Megapool Kreatech reference DNA. This most closely corresponds to the expected situation in the peripheral blood of patients with OC tumor burden. The cftDNA present in the blood will coexist alongside the normal cfDNA and thus represent unknown dilutions. In Figure 4 we show that with the ddPCR assay combination *MRGPRX*(FAM)-*HECW1*(HEX) we can still clearly indicate the genomic amplification up to a dilution of 1:256. The simulation shows that the skillful selection of appropriate ddPCR-CNV assay combinations can indicate genomic imbalances.

As we have already shown, we have developed a detection of OC cells using an OC-FISH probe based on aCGH data (Figure 2) [21]. Using these data, we identified comparable conspicuous loci and generated ddPCR-CNV assays for both conspicuous gains and conspicuous genomic losses (Figure 2). The reference ddPCR-CNV assays (*HECW1* and *USP7*) required for the test combinations, with rather unremarkable genomic loci in OC, have already proven themselves in the simulation. We have expanded these to include *JAK1*. The combination of reference assays and test assays, both of which are colored differently, thus allows for comparative ddPCR. One ddPCR-CNV assay serves as the reference and the second test assay checks for a possible imbalance in simultaneous comparison to the reference. Figure 5 shows all the test combinations that we carried out on the 55 test subjects without a tumor background and 15 patients with OC. As is already clear show in the graphic, there are test combinations that clearly differentiate between patients with OC and subjects without a tumor background. The values of both groups differ significantly in the ddPCR test combinations *JAK1*-*PAK2*, *JAK1*-*PVT1*, *JAK1*-*MYOCD*, *JAK1*-*TIMM21*, *USP7*-*TIMM21*, and *JAK1*-Chr22 in the two-sample *t*-test (Figure 6; Table 1). To assess whether a test combination is abnormal, the data from all subjects without a tumor background were used as a benchmark. For each test combination, the Xs values that lie outside the 25% and 75% percentiles of all probands without a tumor background were to be considered abnormal. The ddPCR supermix used does not perform reverse transcription. Because gene expression products are not detected by the ddPCR, different detection levels of the individual tests can only be attributed to the genomic templates available in the blood. However, cfDNA in the blood is likely to be protected from nucleases to different degrees depending on the chromatin status. This may be a reason for the different presence levels of cfDNA in the templates. Templates from heterochromatic regions are more condensed and protected than those from euchromatic regions. No test combination with *HECW1* is suitable for differentiating between OC and healthy subjects. However, almost all test combinations with *JAK1* are able to do so. *USP7-Timm21* also differentiates significantly. With the exception of *ZFAT*, *PAK2*, and *HECW1*, all the cfDNA homolog regions to be tested originate from heterochromatic chromosome sections and are therefore likely to be packaged in a more protected manner than euchromatic regions. The fact that the test combinations with *ZFAT* and *HECW1* differentiate slightly, but not significantly, between OC and healthy states may be due to faster nucleic acid degradation in the blood.

As Table 2 shows, the proportion of conspicuous test combinations within the cases classified as “true negative” is considerably lower than that within the cases classified as “true positive”. Nevertheless, there are misinterpretations in the classification that lead to incorrect assessments of the cases. The criteria we propose here are not absolutely clear and did lead to incorrect classifications. A cluster analysis of our training cohort (not published here) even found only one “false negative” assessment when taking all test combinations into account. However, the assessment criteria of the cluster analysis are difficult to subject to clear decisions in the context of validation. However, the decision as to whether the Xs value of a tested individuals is within or outside the 25% percentile of a test combination can be decided. This also applies to future validations. The evaluations summarized in Table 2 for the six significantly differentiating ddPCR-CNV test combinations reflect the most correct results when the results of an examined case are outside the 25% and 75% percentiles in at least four test combinations. Using these evaluation criteria, we were able to confirm 12 out of 15 true OC cases with these six comparative ddPCR-CNV assays using cftDNA in the peripheral blood. We were also able to confirm 44 out of 55 subjects without a tumor background as healthy. The test failed to identify three tumor patients with OC as patients with OC. Furthermore, 11 out of 55 otherwise healthy subjects showed abnormalities. This results in a significance of 0.8 and a specificity of 0.8 for the combination of these six tests.

The blood samples from patients with OC were always taken immediately before the surgical procedure. In principle, it can be assumed that the patients had a high tumor burden, which was already causing symptoms. This test is therefore nothing spectacular at first. However, the method presented here basically shows that the typical genomic imbalances of OC can be indicated by cftDNA in the peripheral blood system. Although the simulation results show that OC-CNV detection is still possible even at relatively high dilutions, the dilution factor in patients with OC still remains an unknown. We do not know the actual ratio of cftDNA to cfDNA in the blood of patients with OC. However, our results are clearly still within the possible detection limits indicated in the simulation. Unlike the efforts of Grebnev et al. (2024) [22], as presented here and in a ddPCR user meeting in 2024, we do not assume tumor-relevant genes but instead use recurrent genomic imbalances to generate suitable ddPCR tests [23]. Because there is currently no early detection method for OC, the suitability of the test presented here as an early detection method can only be directly determined in a screening study. However, it can be assumed that the amount of detectable cftDNA correlates with a patient’s tumor load. This tumor load changes in a patient with OC during treatment. In surgery, as much tumor material as possible is removed. Residual tumor material is subsequently treated with chemotherapy. The tumor load therefore changes during therapy. In order to avoid a very extensive screening study, which is certainly necessary, in a much smaller study, we are testing the longitudinal detection possibility of the tumor before surgery, before the start of chemotherapy, after the 3rd cycle of chemotherapy, and four weeks after the end of chemotherapy. This will give us an indication of whether this test is also promising in a screening study. We are hopeful that we will be able to present the results shortly. In view of a potential application in the early detection of OC, it is important to keep the number of “false negative” assessments as low as possible. Such cases would not be treated, even in screening, and without treatment OC threatens to spread. “False positive” findings, on the other hand, would be subjected to further testing, which, without a critical finding, would probably only incur costs but no damage to health. The extent to which this test is suitable for early detection will be shown by the results of the aforementioned longitudinal studies using our test procedure on patients with OC undergoing therapy. If this test can also show a reduced tumor burden after surgery, we can assume that this test procedure can be tested on a separate validation cohort.

The singular argument that we can actually confirm gains or losses in loci where we expect gains or losses based on the aCGH data using the significant ddPCR-CNV assay combinations underlines the functionality of this method. OC-typical losses in loci such as 18q22.32 are confirmed as losses in most patients with OC by the significant ddPCR-CNV combinations using the *Timm21* ddPCR-CNV assay. Even gains that can be expected based on the aCGH data are confirmed as such in most patients with OC by the significantly differentiating ddPCR-CNV assay combinations with *PAK2* (3q29) and *PVT1* (8q24.21) (Figure 6). However, with *MYOCD* in 17p12 and Chr22q13.32, there are also regions that should have losses in 60% of the patients with OC examined using aCGH. Nevertheless, most of the patients with OC examined using our test combinations involving *MYOCD* and Chr22 showed gains rather than losses in these regions. Do these results, which contradict the aCGH results, speak against this method? Not really! The genomic chaos in the OC cells can probably hardly be systematized, even with aCGH methods. As Figure 2 shows, in addition to the 60% losses in 17p12 and 22q13.32, gains of up to 10% are also detected in these regions with aCGH. Admittedly, the assumption that the majority of ddPCR-CGH assay combinations with Chr22 and *MYOCD* are precisely the 10% found in the aCGH is rather unlikely to be incorrect, but not impossible. Perhaps the tactic of selecting the ddPCR-CNV assay combinations based on the aCGH data was also completely irrelevant. Perhaps the genomic chaos in OC cells would have made it possible to simply throw several loci together and then look for tests that significantly discriminate between OC and normal subjects. However, it is important that we found ddPCR-CNV assay combinations that can largely distinguish between sick and non-sick people. Cytogeneticists have long known that solid tumors carry genomic chaos within them [24,25,26]. In particular, 20–30 years ago, the cytogenetics of leukemia [27] defined a hope that the few translocations and gene fusions in leukemic neoplasms, which clearly determine the development of neoplasms, could also be applied to solid tumors [28]. This has not been confirmed, not least because of the chaotic genome state in solid tumors [25]. Nevertheless, with ever-improving techniques that allow us to sequence entire tumor genomes in a very short time, we have continued to focus on tumor-determining and influencing gene mutations and have generated a great deal of knowledge [29,30]. This is also the case in the study of OC [31], as we know full well that earlier detection of OC can probably save the lives of many affected people [32,33]. ddPCR is an available a tool that can detect even the smallest amounts of nucleic acids, down to individual molecules [34]. It is actually quite obvious to compare loci with few changes to those with many changes and thus detect cftDNA. It is unclear whether OC-typical CNVs can still be detected when the ratio is reduced to the detriment of cftDNA. Such a condition can be suspected in patients with OC whose disease is not yet very advanced and who may not even suspect that they have the disease. It, therefore, cannot be ruled out that the test procedure presented here is suitable for detecting OC at an earlier stage. We are currently working on a follow-up study in which we will use this test procedure to monitor the course of therapy. This will give us information on the extent to which the test result changes with complete remission, and with decreasing and possibly increasing tumor load. This may give us an indication of how the potential for OC detection through our test correlates with tumor stage. If these hopes are confirmed in further studies, we might have a tool at our disposal with which we can confirm the suspicion of OC at an earlier stage. This would mean that this test combination is suitable for detecting OC, which is asymptomatic in itself, at an earlier time using the ddPCR-CNV procedure. Further follow-up studies will have to show whether this will also be accompanied by a general reduction in OC mortality.

## 5. Conclusions

Based on typical aCGH results of OC, we have succeeded in developing ddPCR tests that indicate the existence of OC by detecting cftDNA with a significance of 0.8 and a specificity of 0.8. We do not use the detection of specific gene mutations as a biomarker but rather demonstrate the genomic chaos in OC itself. The test combinations *JAK1*-*PAK2*, *JAK1*-*PVT1*, *JAK1*-*MYOCD*, *JAK1*-*TIMM21*, *USP7*-*TIMM21*, and *JAK1*-Chr22 have proven to be significantly differentiating test combinations for detecting OC. If at least four of these six test combinations show an Xs value outside the 25–75% percentile of normal probands without a tumor background, we can assume the existence of OC. The patients with OC tested here can be assumed to have a relatively high tumor burden before surgery. A follow-up study that has already begun will document the change in tumor load during therapy using our tests to show whether these tests are also suitable for detecting lower tumor loads. Only then will it be clear whether this test has the potential to be a screening marker for OC.

## Figures and Tables

**Table 1:** Standard deviations of the determined quotients (Xi) from the droplet numbers of positive ddPCR-CNV assay combinations (Xs).

ddPCR-CNV-Test	Probands w/o Tumor			Patients with OC			
FAM- HEX	Standard Deviation	75% Percentile Xs	25% Percentile Xs	Standard Deviation	75% Percentile Xs	25% Percentile Xs	*t*-Test
HECW1-ZFAT	0.08136	0.51390	−0.81022	0.12473	2.08208	−0.00890	0.01149
JAK1-ZFAT	0.18390	0.24363	−0.61416	0.23164	1.32519	0.11473	0.01458
USP7-ZFAT	0.07160	0.37532	−0.62811	0.15354	0.32901	−1.31674	0.07989
HECW1-PAK2	0.09099	0.69514	−0.62591	0.14927	1.28455	−0.04860	0.06441
JAK1-PAK2	0.16983	0.45317	−0.81575	0.20707	2.56683	0.85132	0.00017
USP7-PAK2	0.05319	0.52323	−0.70487	0.05203	1.17016	−0.06223	0.09456
HECW1-PVT1	0.08764	0.56085	−0.77935	0.09571	1.20560	−0.46190	0.15315
JAK1-PVT1	0.18017	0.60500	−0.75684	0.20353	1.83776	0.09832	0.00124
USP7-PVT1	0.10433	0.37627	−0.54911	0.12323	0.48061	−1.34183	0.18800
HECW1-MYOCD	0.08915	0.33484	−0.67082	0.18493	1.44937	−0.15328	0.32778
JAK1-MYOCD	0.21583	0.57897	−0.73360	0.23197	1.34405	0.31920	0.00384
USP7-MYOCD	0.07858	0.38206	−0.54142	0.09742	0.50998	−0.50520	0.81465
HECW1-TIMM21	0.16109	0.67275	−0.72299	0.19251	−0.22700	−1.77490	0.01931
JAK1-TIMM21	0.13145	0.68048	−0.55552	0.13140	−0.20390	−1.49240	0.00866
USP7-TIMM21	0.16763	0.71718	−0.85622	0.20159	−0.70560	−2.13100	0.00236
HECW1-Chr22	0.08796	0.79017	−0.78422	0.09996	1.05194	0.20039	0.02901
JAK1-Chr22	0.25401	0.15682	−0.55148	0.20903	1.47322	0.30466	0.00179
USP7-Chr22	0.09853	0.39274	−0.46181	0.08671	0.47467	−0.85511	0.78677

The two-sample *t*-test indicates which ddPCR-CNV assay combinations can detect significant differences between subjects without tumors and patients with OC when *t* < 0.01. The data highlighted in yellow correspond to the results of subjects without a tumor background. The data highlighted in pink correspond to the results of patients with ovarian cancer. The tests in the test combinations written in blue are marked with the fluorophore FAM and the tests in the test combinations written in green are marked with the fluorophore Hex.

**Table 2:** The number of correctly and incorrectly classified cases within the test cohort is listed at the top. The proportions of abnormal test combinations for each determined class are also given. The patients with OC are listed below and compared with the determined classifications and the results of the individual test combinations. Abnormal test combinations are shown with an “x” against a yellow background and unremarkable test combinations are shown with a “-” against a white background.

	Number of Cases	JAK1-PAK2	JAK1-PVT1	JAK1-MYOCD	JAK1-TIMM21	USP7-TIMM21	JAK1-Chr22
true negative	44	16/44 (36%)	16/44 (36%)	7/44 (16%)	18/44 (41%)	9/44 (20)	8/44 (18)
true positive	12	12/12 (100%)	10/12 (83%)	11/12 (92%)	9/12 (75%)	11/12 (92%)	12/12 (100%)
false negative	3	1/3 (33%)	1/3 (33%)	0/3 (0%)	0/3 (0%)	2/3 (66%)	1/3 (33%)
false positive	11	11/11 (100%)	10/11 (91%)	6/11 (55%)	10/11 (91%)	5/11 (45%)	6/11 (55%)
OC-Cases	Classification						
OC-Patient 06	true positive	x	-	-	x	x	x
OC-Patient 10	true positive	x	x	x	x	x	x
OC-Patient 17	true positive	x	x	x	-	x	x
OC-Patient 26	true positive	x	x	x	-	-	x
OC-Patient 39	false negative	-	-	-	-	x	-
OC-Patient 51	false negative	x	x	-	-	-	x
OC-Patient 54	false negative	-	-	-	-	x	-
OC-Patient 62	true positive	x	x	x	x	x	x
OC-Patient 63	true positive	x	x	x	x	x	x
OC-Patient 65	true positive	x	x	x	x	x	x
OC-Patient 67	true positive	x	x	x	x	x	x
OC-Patient 69	true positive	x	x	x	-	x	x
OC-Patient 70	true positive	x	x	x	x	x	x
OC-Patient 73	true positive	x	x	x	x	x	x
OC-Patient 84	true positive	x	-	x	x	x	x

In the test combinations, tests marked with the fluorophore FAM are labeled blue and those marked with the fluorophore HEX are labeled green. Correctly assessed findings are highlighted in green and incorrectly assessed findings are highlighted in red. Red labels correspond to real tumors and green labels correspond to subjects without tumors. Findings highlighted in yellow correspond to abnormal results (x) and results highlighted in white are unremarkable (-).

## Data Availability

The data presented in this study are available on request from the corresponding author.

## Supplementary Materials

The following supporting information can be downloaded at https://www.mdpi.com/article/10.3390/cancers17050780/s1, Table S1: Xs values of blood samples tested with significantly differentiating ddPCR-CNV assay combinations; Table S2: Analysis of Variance (ANOVA) of all test combinations applied to the test cohort (n = 70 patients); Table S3: Cluster analysis of all test combinations applied to all 70 patients. Within the training cohort, only patient 54 was classified as a false negative.
