# Supplementary material for: Detection of Genomic Copy Number Variations in Ovarian Cancer in the Peripheral Blood System
Source: Cancers (Basel). 2025 Feb 25;17(5):780. doi: 10.3390/cancers17050780 (PMC11898772; doi:10.3390/cancers17050780)
Supplement: Supplementary file 1 [file cancers-17-00780-s001.zip › Table S1.pdf]

Supplement Table S1. Xs values of blood samples tested with significantly differentiating ddPCR-CNV assay combinations.

	JAK1-PAK2 [Xs]	JAK1-PVT1 [Xs]	JAK1-MYOCD [Xs]	JAK1-TIMM21 [Xs]	USP7-TIMM21 [Xs]	JAK1-Chr22 [Xs]	
Patient 01	0.34472	0.60500	-0.07786	-0.67687	-0.98428	-0.33591	true negative
Patient 02	0.03897	0.26689	-0.47852	-0.59725	-1.10819	-0.07201	true negative
Patient 03	-0.54824	-1.09603	-0.65480	0.73854	0.79263	-0.46962	true negative
Patient 04	2.06049	1.57172	0.60649	-0.21248	0.06494	2.28042	true negative
Patient 05	0.79200	0.98413	2.04331	-0.96238	-1.02951	-0.08747	false positive
OC-Patient 06	1.35448	-0.06758	0.23158	-1.55126	-1.58755	0.28723	true positive
Patient 07	1.42553	1.61583	2.01757	-0.16656	-1.48787	0.71394	true negative
Patient 08	-0.72834	-1.53839	-0.71624	1.33238	0.44793	-0.56003	true negative
Patient 09	-0.80354	-0.54505	-0.52565	1.56693	0.70141	-0.64282	true negative
OC-Patient 10	2.47553	1.70427	2.39983	-2.45842	-1.83623	1.48792	true positive
Patient 11	-1.12319	-0.56276	-1.01459	0.86616	1.33181	-0.55148	true negative
Patient 12	-0.15746	-0.55924	0.28100	-0.00161	0.60261	0.04326	true negative
Patient 13	0.36760	0.39074	0.62115	-0.58227	-0.85622	0.15654	true negative
Patient 14	-1.03819	-1.27682	-0.90285	1.27690	1.54033	-0.96837	false positive
Patient 15	-1.04299	0.08746	-0.97596	-0.17403	-0.42840	-0.69803	true negative
Patient 16	0.42125	0.59722	0.57897	-0.85052	-1.14932	0.37288	true negative
OC-Patient 17	1.77802	1.60651	0.82805	0.33857	-1.45175	1.94991	true positive
Patient 18	-0.05085	0.42660	0.72729	0.69970	0.02626	-0.13119	true negative
Patient 19	-0.06631	0.60684	0.38334	-0.55552	-0.89292	-0.08774	true negative
Patient 20	-0.52175	-1.03466	-0.89783	0.47603	1.06196	-0.31809	true negative
Patient 21	2.22466	2.53856	2.22759	-1.56042	-1.56786	0.71471	false positive
Patient 22	-1.43037	-0.98919	-0.84695	-0.23717	0.55671	-0.50766	true negative
Patient 23	-0.82311	-0.44370	-0.34990	0.05964	0.10193	-0.28180	true negative
Patient 24	2.33087	1.38394	1.52151	-2.55334	-2.31032	-0.05046	false positive
Patient 25	-1.01497	-0.99288	-0.84114	0.88376	1.09804	-0.91036	false positive
OC-Patient 26	1.34412	1.97914	1.06119	-0.15929	-0.53221	0.32209	true positive
Patient 27	1.50367	1.87350	1.31698	-0.68174	-2.13492	0.90958	false positive
Patient 28	-0.82408	-0.60524	-0.78572	0.89118	0.81167	-0.81687	false positive
Patient 29	-0.86110	-0.18399	-0.24776	0.46297	-0.29346	0.08140	true negative
Patient 30	-0.09145	-0.03056	-0.43873	0.86749	0.36364	-0.18083	true negative
Patient 31	-0.51646	-0.46953	-0.32698	0.41571	0.91942	-0.16454	true negative
Patient 32	0.42264	0.50191	0.63815	0.03660	-0.38992	0.36424	true negative
Patient 33	-0.17231	0.60571	0.10618	-0.33793	-1.07077	0.06279	true negative
Patient 34	-1.07014	-1.51706	-1.20035	1.02085	1.46811	-1.05322	false positive
Patient 35	-1.05515	-0.76625	-0.73360	0.68048	0.90925	-0.59819	true negative
Patient 36	0.49083	1.48594	0.63852	-0.20342	-0.48731	0.75784	true negative
Patient 37	0.79463	0.53940	0.10901	0.54166	-0.19490	0.44418	true negative
OC-Patient 39	-0.48479	0.10425	-0.02309	-0.24870	-0.87904	0.07464	false negative
Patient 41	0.59430	0.89588	0.43504	0.06270	-0.51127	0.19411	true negative
Patient 42	-0.81575	-1.06793	-1.16512	1.59684	1.73845	-0.96689	false positive
Patient 43	-0.59581	-1.07580	-0.91363	0.67369	0.89587	-0.94950	true negative
Patient 44	-0.58296	-1.68012	-0.57877	0.40284	0.66707	-0.61513	true negative
Patient 45	-0.38518	0.21695	-0.47975	0.01601	0.52590	-0.04977	true negative
Patient 46	-0.66358	0.07801	-0.29844	1.23285	0.29473	-0.31952	true negative
Patient 47	-0.94614	-0.76360	-0.22781	1.10651	0.52082	-0.65836	true negative

Patient 49	-0.48209	-0.69469	-0.45785	0.18793	0.44609	-0.21745	true negative
OC-Patient 51	0.89530	1.00745	0.40682	-0.44645	-0.47597	0.43821	false negative
Patient 52	0.45317	-0.61031	-0.53142	-0.50154	-0.41340	0.14506	true negative
Patient 53	0.31784	0.53988	0.52336	-0.61892	-0.71854	0.15682	true negative
OC-Patient 54	-0.34406	0.09240	-0.21784	0.38502	1.43892	-0.04367	false negative
Patient 55	0.45051	1.36720	0.41370	-0.50325	-1.15309	1.68602	true negative
Patient 57	0.55053	0.06611	-0.46320	-0.67656	-0.09687	-0.46624	true negative
Patient 58	-0.80854	-0.59690	-0.43124	0.59457	0.79213	-0.36995	true negative
Patient 59	-0.09098	-0.03367	-0.66676	-0.25942	-0.07109	-0.43067	true negative
Patient 60	2.58481	-0.49049	0.94883	-4.43064	1.42655	0.23705	true negative
Patient 61	-0.88240	-0.55564	-0.81675	-0.28391	0.31994	-0.11060	true negative
OC-Patient 62	2.62072	1.97125	3.07703	-1.08695	-1.56010	2.17400	true positive
OC-Patient 63	1.73146	1.06339	1.07329	-0.80234	-1.24264	0.68055	true positive
Patient 64	-0.40641	-0.75684	-0.77154	0.75414	0.92907	-0.46451	true negative
OC-Patient 65	2.89768	1.12372	0.59355	-2.43739	-2.65577	1.19321	true positive
Patient 66	-1.19503	-0.96261	-1.14257	0.28867	0.71172	-0.79837	false positive
OC-Patient 67	2.76897	1.69519	1.37046	-1.99848	-2.40482	1.22404	true positive
OC-Patient 69	0.80733	1.56337	0.64010	-0.27948	-1.39885	0.78372	true positive
OC-Patient 70	2.65163	2.70899	1.31764	-0.89372	-2.08978	1.67782	true positive
Patient 71	-0.14227	-0.97445	-0.75862	0.36926	0.43897	-0.63821	true negative
OC-Patient 73	2.06241	3.09514	2.60709	-0.86226	-2.20549	1.45853	true positive
Patient 74	-0.53362	-0.55212	-0.50046	-0.14448	0.71784	-0.21104	true negative
Patient 81	0.47030	0.90806	1.01415	0.31603	-0.42559	0.99323	true negative
Patient 82	1.60949	1.19731	1.63956	-0.94353	-1.01326	1.02557	false positive
OC-Patient 84	2.51294	0.05805	1.16062	-1.43357	-2.17241	0.72938	true positive

In the test combinations, tests marked with the fluorophore FAM are labeled blue and those marked with the fluorophore HEX are labeled green. Correctly assessed findings are highlighted in green and incorrectly assessed findings are highlighted in red. Red labels correspond to real tumors and green labels correspond to subjects without tumors. Findings highlighted in yellow correspond to abnormal results (Xs) and results highlighted in white are unremarkable.
