# Supplementary material for: Detection of Genomic Copy Number Variations in Ovarian Cancer in the Peripheral Blood System
Source: Cancers (Basel). 2025 Feb 25;17(5):780. doi: 10.3390/cancers17050780 (PMC11898772; doi:10.3390/cancers17050780)
Supplement: Supplementary file 1 [file cancers-17-00780-s001.zip › Table S2.pdf]

Supplement Table S2: Analysis of Variance (ANOVA) of all test combinations applied to the test cohort (n=70 patients). SS = sum of squares; df= degree of freedom; F = ratio of the mean square for the between groups divided by the mean square within groups; p = less than 0,05 means: The hypothesis that the test combination is not differentiated into two clusters is rejected.

	ANOVA for continuous variables (Xs Diskriminanz 31-01-2025)<br>Number of clusters: 2<br>Total number of training cases: 70					
	betwee SS	df	within SS	df	F	p value
HECW1-ZFAT	31.59995	1	72.4238	68	29.6698	0.000001
JAK1-ZFAT	28.87112	1	38.2459	68	51.3320	0.000000
USP7-ZFAT	12.70017	1	118.3280	68	7.2985	0.008705
HECW1-PAK2	28.90758	1	74.6040	68	26.3487	0.000003
JAK1-PAK2	61.94601	1	39.9138	68	105.5357	0.000000
USP7-PAK2	0.36492	1	68.8815	68	0.3602	0.550365
HECW1-PVT1	9.75466	1	55.0986	68	12.0387	0.000909
JAK1-PVT1	34.17138	1	51.2553	68	45.3349	0.000000
USP7-PVT1	0.80503	1	70.8226	68	0.7729	0.382405
HECW1-MYOCD	15.29912	1	99.8280	68	10.4213	0.001919
JAK1-MYOCD	32.53254	1	38.3752	68	57.6470	0.000000
USP7-MYOCD	0.42843	1	70.0613	68	0.4158	0.521200
HECW1-TIMM21	28.64230	1	49.0532	68	39.7054	0.000000
JAK1-TIMM21	30.04095	1	46.7212	68	43.7228	0.000000
USP7-TIMM21	39.93927	1	48.2560	68	56.2805	0.000000
HECW1-Chr22	22.43143	1	54.5361	68	27.9693	0.000001
JAK1-Chr22	15.30718	1	28.2281	68	36.8741	0.000000
USP7-Chr22	1.78508	1	65.6661	68	1.8485	0.178447
