# Supplementary material for: Detection of Genomic Copy Number Variations in Ovarian Cancer in the Peripheral Blood System
Source: Cancers (Basel). 2025 Feb 25;17(5):780. doi: 10.3390/cancers17050780 (PMC11898772; doi:10.3390/cancers17050780)
Supplement: Supplementary file 1 [file cancers-17-00780-s001.zip › Table S3.pdf]

Supplement Table S3: Cluster analysis of all test combinations applied to all 70 patients. Within the training cohort, only patient 54 was classified as a false negative.

Cluster members (Xs Diskriminanz 31-01-2025)
Number of clusters: 2
Total number of training cases: 70

Case Name	Case No	Final Classification	OC	HECW1-ZFAT	JAK1-ZFAT	USP7-ZFAT	HECW1-PAK2	JAK1-PAK2	USP7-PAK2	HECW1-PVT1	JAK1-PVT1	USP7-PVT1	HECW1-MYOCD	JAK1-MYOCD	USP7-MYOCD	HECW1-TIMM21	JAK1-TIMM21	USP7-TIMM21	HECW1-Chr22	JAK1-Chr22	USP7-Chr22	Distance to centroid
Patient 01	1	1	0	0.70037	0.10694	1.33864	-1.32068	0.34472	-0.79447	2.58078	0.60500	-0.10421	-0.07524	-0.07786	1.29627	-0.08135	-0.67687	-0.98428	0.74713	-0.33591	-1.52724	0.941146
Patient 02	2	1	0	-1.30068	-1.30019	-0.01922	0.64405	0.03897	-0.30310	0.03138	0.26689	1.78466	-0.33736	-0.47852	-0.54142	-0.24980	-0.59725	-1.10819	1.19996	-0.07201	-0.96100	0.686557
Patient 03	3	1	0	-0.80898	-0.58847	-0.52091	-0.90094	-0.54824	1.05588	-0.90064	-1.09603	-0.20883	-1.04130	-0.65480	0.23060	0.53435	0.73854	0.79263	-1.08013	-0.46962	-0.41347	0.480438
Patient 04	4	1	0	-0.08830	0.24363	-1.62621	-0.29284	2.06049	-0.85357	0.20246	1.57172	1.33699	0.59987	0.60649	1.59566	-0.84418	-0.21248	0.06494	0.92280	2.28042	4.31564	1.322214
Patient 05	5	1	0	2.60563	0.74439	0.26744	-0.93060	0.79200	-1.50728	0.06594	0.98413	2.27193	0.31107	2.04331	0.32417	-1.33417	-0.96238	-1.02951	0.91794	-0.08747	2.10192	1.158977
Patient 06	6	2	1	1.47068	-0.36724	0.64098	1.25098	1.35448	0.61082	-0.95558	-0.06758	-1.55289	0.61510	0.23158	-0.79543	-1.30469	-1.55126	-1.58755	0.38026	0.28723	-0.62561	0.782037
Patient 07	7	2	0	2.44339	1.04505	-0.10085	1.70719	1.42553	-0.24396	1.63543	1.61583	-0.38073	-1.41302	2.01757	-0.58433	-1.33669	-0.16656	-1.48787	3.39426	0.71394	1.75518	1.252735
Patient 08	8	1	0	0.57436	-0.40807	2.07313	0.69514	-0.72834	2.16881	-1.21348	-1.53839	-1.28116	-0.32249	-0.71624	-2.04162	1.50316	1.33238	0.44793	-0.89912	-0.56003	-0.82750	0.874948
Patient 09	9	1	0	-0.88123	-0.28271	0.20211	-0.31883	-0.80354	0.03897	-1.30606	-0.54505	-0.24329	-0.71089	-0.52565	0.25836	1.11725	1.56693	0.70141	-0.34950	-0.64282	0.38278	0.510581
Patient 10	10	2	1	-0.03748	3.97916	-0.69256	-0.76628	2.47553	0.95509	-0.54282	1.70427	-0.40469	-0.94408	2.39983	-0.22728	-1.80931	-2.45842	-1.83623	3.49787	1.48792	-0.55525	0.992154
Patient 11	11	1	0	0.13412	-0.83365	1.45894	-1.15865	-1.12319	0.41115	-0.32028	-0.56276	-0.47560	-0.74079	-1.01459	0.67034	1.26870	0.86616	1.33181	-0.49790	-0.55148	-0.31151	0.568608
Patient 12	12	1	0	0.09438	0.15387	-0.49032	-1.79001	-0.15746	-0.48156	-0.18348	-0.55924	-0.11656	0.69963	0.28100	-0.78212	0.11560	-0.00161	0.60261	-0.22313	0.04326	1.01583	0.400117
Patient 13	13	1	0	1.31844	0.15788	-0.12192	-0.54744	0.36760	-1.14327	1.61436	0.39074	0.37627	0.65228	0.62115	0.58829	-0.81701	-0.58227	-0.85622	1.31776	0.15654	0.29507	0.811490
Patient 14	14	1	0	-0.95810	-1.05129	-0.86428	-0.81514	-1.03819	0.68461	-0.46001	-1.27682	-0.60159	-0.74096	-0.90285	-0.34963	1.52230	1.27690	1.54033	-0.84065	-0.96837	-0.46181	0.704564
Patient 15	15	1	0	-0.88227	-0.56441	-0.61879	1.34455	-1.04299	-0.00783	-0.66575	0.08746	0.15969	0.17724	-0.97596	1.77445	0.58986	-0.17403	-0.42840	-1.40933	-0.69803	-0.43412	0.614097
Patient 16	16	1	0	0.14405	1.40428	0.64598	-0.74884	0.42125	0.17738	-0.56440	0.59722	1.24069	0.78723	0.57897	0.26416	-0.72299	-0.85052	-1.14932	-0.81231	0.37288	0.71980	0.711063
Patient 17	17	2	1	0.01969	1.08871	0.66700	0.12096	1.77802	1.56422	1.25845	1.60651	0.66928	0.35348	0.82805	0.44483	-0.98217	0.33857	-1.45175	0.77367	1.94991	1.28155	0.691921
Patient 18	18	1	0	-1.36406	0.33655	-1.02203	-1.06132	-0.05085	-1.10213	-0.39149	0.42660	-0.54911	-0.35283	0.72729	2.00456	0.66067	0.69970	0.02626	0.34919	-0.13119	-0.45856	0.604171
Patient 19	19	1	0	1.14502	0.24063	-0.29116	0.93063	-0.06631	0.52323	0.94340	0.60684	0.15722	0.07854	0.38334	1.25804	-1.21110	-0.55552	-0.89292	-1.53253	-0.08774	0.47615	0.729730
Patient 20	20	1	0	0.30359	-0.54920	0.06734	0.30061	-0.52175	-0.32206	0.50696	-1.03466	-1.01783	-0.55890	-0.89783	-0.51957	0.73396	0.47603	1.06196	-0.78422	-0.31809	-1.18142	0.508261
Patient 21	21	2	0	-0.84579	1.28775	-0.60567	0.12236	2.22466	0.85342	-0.56866	2.53856	1.43046	2.24001	2.22759	2.50763	-1.56239	-1.56042	-1.56786	1.19506	0.71471	0.61748	1.310339
Patient 22	22	1	0	-0.73746	-0.86558	-0.99963	0.75314	-1.43037	-0.01091	-0.87853	-0.98919	-0.00985	1.04890	-0.84695	-0.81404	1.53557	-0.23717	0.55671	-0.14867	-0.50766	-0.05712	0.603004

Patient 23	23	1	0	-0.84595	0.06601	-0.41289	-0.33603	-0.82311	0.08080	0.29551	-0.44370	-0.13209	0.10416	-0.34990	-0.36601	-0.00232	0.05964	0.10193	-0.65684	-0.28180	0.46129	0.280299
Patient 24	24	2	0	1.03369	2.02046	0.60442	1.45845	2.33087	-1.56775	0.90208	1.38394	-0.07617	0.02568	1.52151	-0.11487	-1.53390	-2.55334	-2.31032	-0.77761	-0.05046	-0.14894	1.223229
Patient 25	25	1	0	0.30687	-0.87538	-0.00011	-0.15564	-1.01497	0.77374	-0.25590	-0.99288	0.24905	-0.55094	-0.84114	-0.52192	1.15974	0.88376	1.09804	-1.25825	-0.91036	-0.52399	0.575402
Patient 26	26	2	1	1.91868	0.28645	1.59339	-0.05032	1.34412	-0.19159	1.95134	1.97914	1.02732	0.47772	1.06119	0.29245	-0.39146	-0.15929	-0.53221	0.52121	0.32209	0.26686	0.731704
Patient 27	27	2	0	1.01636	1.48603	-0.62811	1.40748	1.50367	0.04562	2.12954	1.87350	2.19925	2.15120	1.31698	2.19686	-1.47554	-0.68174	-2.13492	1.82491	0.90958	-2.39856	1.301550
Patient 28	28	1	0	0.13037	-1.07356	-0.00376	0.70147	-0.82408	-0.55922	-0.11658	-0.60524	-0.67956	-1.14771	-0.78572	0.11556	0.64495	0.89118	0.81167	0.21653	-0.81687	-0.16644	0.463124
Patient 29	29	1	0	-0.46940	-0.05737	0.46451	-0.40272	-0.86110	1.32330	-0.52482	-0.18399	0.29094	-1.26313	-0.24776	0.28014	0.21191	0.46297	-0.29346	0.79017	0.08140	0.03285	0.403253
Patient 30	30	1	0	-0.89261	-0.02053	0.37532	-0.95620	-0.09145	0.80490	-0.02297	-0.03056	-0.16609	1.48610	-0.43873	0.61184	0.59059	0.86749	0.36364	-0.08633	-0.18083	0.36683	0.343080
Patient 31	31	1	0	-0.68700	-0.63090	-0.65720	-0.51349	-0.51646	-0.46649	-0.74676	-0.46953	-0.37880	-0.35405	-0.32698	-1.62882	0.52262	0.41571	0.91942	-1.63580	-0.16454	-0.83992	0.524528
Patient 32	32	1	0	-0.44303	-0.13207	-0.76875	-0.07676	0.42264	0.46181	-0.01242	0.50191	-0.22390	-0.34486	0.63815	1.73691	-0.60418	0.03660	-0.38992	0.42799	0.36424	0.41177	0.541021
Patient 33	33	1	0	0.12572	-0.38860	2.36694	0.48259	-0.17231	0.50686	0.11622	0.60571	0.00105	0.69657	0.10618	-1.25633	-0.75453	-0.33793	-1.07077	-0.69728	0.06279	0.00043	0.584789
Patient 34	34	1	0	-1.22895	-1.07804	-0.29868	-0.58044	-1.07014	0.42773	-1.06095	-1.51706	-0.36301	-0.47404	-1.20035	-0.48618	0.95074	1.02085	1.46811	-0.97145	-1.05322	-0.36517	0.722196
Patient 35	35	1	0	-0.86292	-0.93929	0.27104	-0.56269	-1.05515	-0.82991	-0.87848	-0.76625	-0.19389	-0.74852	-0.73360	0.38206	0.67275	0.68048	0.90925	-0.78199	-0.59819	0.26039	0.490906
Patient 36	36	1	0	-0.55334	0.64404	-0.73847	-0.57244	0.49083	-0.70487	0.57508	1.48594	-0.25550	0.47308	0.63852	-0.05065	-0.28894	-0.20342	-0.48731	0.34165	0.75784	0.01253	0.653825
Patient 37	37	1	0	-0.29069	0.09563	-0.42328	0.18678	0.79463	3.36166	0.90228	0.53940	-1.84787	-0.41099	0.10901	0.06046	-0.22384	0.54166	-0.19490	0.86678	0.44418	0.35350	0.866536
Patient 39	38	2	1	0.45719	0.62242	0.45898	0.17697	-0.48479	1.42811	-0.95512	0.10425	-0.99047	1.35970	-0.02309	-0.56490	-0.92664	-0.24870	-0.87904	0.83453	0.07464	0.83857	0.973581
Patient 41	39	1	0	-0.95543	-0.69815	-1.44871	0.14818	0.59430	1.90568	0.81204	0.89588	1.10511	0.59318	0.43504	-0.24981	-0.38057	0.06270	-0.51127	0.91168	0.19411	0.86393	0.721450
Patient 42	40	1	0	-0.47051	-1.11332	-0.08927	-0.74181	-0.81575	-0.61487	-0.77935	-1.06793	-0.48157	-1.00532	-1.16512	-1.02436	1.74955	1.59684	1.73845	-0.97833	-0.96689	-0.07129	0.774853
Patient 43	41	1	0	-0.49800	-0.78435	0.34656	-0.05052	-0.59581	0.32040	-1.23618	-1.07580	-0.62584	-0.39218	-0.91363	-0.62391	1.17052	0.67369	0.89587	-0.47173	-0.94950	-0.13432	0.565892
Patient 44	42	1	0	0.17076	-0.65172	-0.41195	0.18599	-0.58296	0.74999	-0.93729	-1.68012	-0.94326	0.26318	-0.57877	-0.80556	0.79691	0.40284	0.66707	-1.01618	-0.61513	-0.24742	0.552697
Patient 45	43	1	0	-0.73647	-0.18408	1.60193	-0.81981	-0.38518	1.26058	-1.24678	0.21695	-0.46773	-0.64688	-0.47975	0.74736	0.45355	0.01601	0.52590	-0.22055	-0.04977	1.40350	0.507832
Patient 46	44	1	0	-0.03906	0.64422	-0.68777	0.74406	-0.66358	-1.50115	0.25981	0.07801	1.79589	-0.00876	-0.29844	0.00597	0.66723	1.23285	0.29473	0.84345	-0.31952	-0.40667	0.616674
Patient 47	45	1	0	-1.21212	-0.52649	-0.38713	-1.29065	-0.94614	-0.32589	-0.86948	-0.76360	0.65544	-0.84782	-0.22781	-0.09844	0.31041	1.10651	0.52082	-0.06919	-0.65836	-0.48119	0.469959
Patient 49	46	1	0	0.34704	-0.42088	1.61395	-0.62591	-0.48209	-0.89700	-0.19412	-0.69469	0.34731	-0.98512	-0.45785	-0.17007	0.76116	0.18793	0.44609	-0.07327	-0.21745	-0.40346	0.362085
Patient 51	47	2	1	0.54563	-0.01482	-1.25154	-0.04697	0.89530	1.75535	0.49536	1.00745	-1.75276	0.27371	0.40682	3.50280	-0.06261	-0.44645	-0.47597	-0.08530	0.43821	-1.78877	0.981921
Patient 52	48	1	0	0.69291	-0.34023	1.48060	0.74464	0.45317	1.14559	-1.09766	-0.61031	-0.63914	-0.80419	-0.53142	0.20855	-0.44950	-0.50154	-0.41340	1.35492	0.14506	0.39274	0.615490
Patient 53	49	1	0	0.55993	0.17362	0.49851	0.88163	0.31784	-0.74443	0.56085	0.53988	1.08515	0.28994	0.52336	0.27530	-0.94989	-0.61892	-0.71854	0.52723	0.15682	-0.52575	0.636718
Patient 54	50	1	1	0.10392	-0.32144	-1.05647	-0.04073	-0.34406	0.74167	-0.29712	0.09240	-0.50147	-0.08336	-0.21784	0.54496	0.06298	0.38502	1.43892	-0.45146	-0.04367	-0.49297	1.069287
Patient 55	51	1	0	0.40889	0.12327	-1.92397	0.20521	0.45051	-0.24858	1.10749	1.36720	-0.50043	0.36242	0.41370	0.65370	-0.70746	-0.50325	-1.15309	0.16119	1.68602	0.03261	0.903368

Patient 57	52	1	0	0.51389	0.09888	-1.64284	0.13902	0.55053	1.40696	0.53106	0.06611	-0.64217	0.24453	-0.46320	-0.84694	-0.30431	-0.67656	-0.09687	-0.31786	-0.46624	-0.87503	0.538761
Patient 58	53	1	0	0.30648	-0.47991	1.24333	-0.30101	-0.80854	-1.01150	-0.39496	-0.59690	-0.85792	-0.46117	-0.43124	-0.73601	0.58453	0.59457	0.79213	-0.78981	-0.36995	-0.15949	0.430620
Patient 59	54	1	0	-0.27400	-0.25125	0.17264	0.95051	-0.09098	0.23793	-1.27983	-0.03367	0.48038	0.33484	-0.66676	0.32415	0.23541	-0.25942	-0.07109	-0.56284	-0.43067	-0.56114	0.413349
Patient 60	55	2	0	3.47891	0.88856	-2.07427	4.50724	2.58481	-0.39702	0.02529	-0.49049	-2.90865	3.91495	0.94883	-1.28693	-2.32616	-4.43064	1.42655	1.40899	0.23705	-2.37834	1.719984
Patient 61	56	1	0	1.06139	-0.24133	0.32713	-0.25582	-0.88240	-0.58292	0.65427	-0.55564	-0.58472	-0.47440	-0.81675	-0.43388	0.07216	-0.28391	0.31994	0.60151	-0.11060	1.11243	0.475402
Patient 62	57	2	1	1.16050	1.15538	-0.43821	-0.64909	2.62072	-0.57602	2.60272	1.97125	0.68935	-0.22320	3.07703	0.94572	-1.80089	-1.08695	-1.56010	0.26925	2.17400	-0.68129	0.910661
Patient 63	58	2	1	2.24548	0.75369	0.19904	1.31812	1.73146	1.36958	0.97075	1.06339	0.29194	-5.19800	1.07329	0.33838	2.05220	-0.80234	-1.24264	0.25419	0.68055	0.87781	1.065387
Patient 64	59	1	0	-0.64660	-0.61416	-0.54950	-0.60368	-0.40641	0.07488	-1.24042	-0.75684	-0.94667	-0.67082	-0.77154	-0.10006	0.79892	0.75414	0.92907	-0.52248	-0.46451	-0.30236	0.473390
Patient 65	60	2	1	4.61923	2.64418	-1.40555	4.93482	2.89768	0.64539	0.78005	1.12372	-1.13077	4.46382	0.59355	-2.19526	-2.24238	-2.43739	-2.65577	0.51445	1.19321	-1.02893	1.142758
Patient 66	61	1	0	0.93319	-0.45845	-0.69678	-0.18479	-1.19503	-1.48186	-0.58425	-0.96261	-1.24490	-0.00513	-1.14257	-0.39064	0.54799	0.28867	0.71172	-0.66883	-0.79837	0.46501	0.616236
Patient 67	62	2	1	2.57880	2.22902	-0.92739	1.56343	2.76897	0.06712	0.07985	1.69519	-1.79684	2.09921	1.37046	-0.44563	-2.02591	-1.99848	-2.40482	1.47242	1.22404	-0.63910	0.612414
Patient 69	63	2	1	0.15460	1.16466	-0.77431	0.66621	0.80733	0.35246	0.49031	1.56337	0.26973	0.84770	0.64010	0.85245	-0.87948	-0.27948	-1.39885	0.66137	0.78372	0.32247	0.458263
Patient 70	64	2	1	-0.05739	0.76169	-6.64967	0.35014	2.65163	-0.42969	-0.38106	2.70899	1.59653	1.53903	1.31764	-0.20672	-1.17417	-0.89372	-2.08978	1.26935	1.67782	0.62687	0.957023
Patient 71	65	1	0	-0.95248	-0.83229	0.23420	-0.49720	-0.14227	-0.08348	-0.57078	-0.97445	-0.52504	-0.65838	-0.75862	0.42286	0.47085	0.36926	0.43897	-0.89811	-0.63821	0.10331	0.386152
Patient 73	66	2	1	-0.17109	0.24429	-5.41835	0.76897	2.06241	-2.10676	1.50438	3.09514	-0.24637	0.99438	2.60709	0.47500	-1.74908	-0.86226	-2.20549	0.14659	1.45853	-1.28904	0.964685
Patient 74	67	1	0	0.05625	-0.55209	0.30737	-0.46513	-0.53362	-0.52878	-0.81588	-0.55212	0.42155	-0.22204	-0.50046	0.04099	0.71539	-0.14448	0.71784	-1.05252	-0.21104	0.41355	0.361509
Patient 81	68	1	0	-0.87799	0.35809	1.19734	-0.69111	0.47030	1.48237	-0.10012	0.90806	0.48587	0.11229	1.01415	-0.71235	-0.16191	0.31603	-0.42559	-0.29748	0.99323	-0.25583	0.676441
Patient 82	69	1	0	-0.41992	1.96043	0.25536	-0.73625	1.60949	-0.63991	1.32967	1.19731	0.18086	-0.89853	1.63956	-0.34193	-1.68599	-0.94353	-1.01326	0.27918	1.02557	-0.53734	1.125382
Patient 84	70	2	1	3.82940	1.48572	-1.38194	4.40319	2.51294	0.58220	1.15276	0.05805	-2.77596	3.03512	1.16062	-1.50034	-1.34403	-1.43357	-2.17241	2.80136	0.72938	-1.12693	1.020392

DC 0 = Patient without tumor; DC 1 = Patient with ovarian cancer.
